# Zoogonids (trematoda) infecting Indo-West Pacific damselfishes (Pomacentridae), including the proposal of a new genus and two new species

**DOI:** 10.1017/S0031182025000307

**Published:** 2025-04

**Authors:** Berilin Duong, Storm B. Martin, Scott C. Cutmore, Thomas H. Cribb

**Affiliations:** 1 School of the Environment, The University of Queensland, St Lucia, QL 4072, Australia; 2 Centre for Sustainable Aquatic Ecosystems, Harry Butler Institute, Murdoch University, Murdoch, WA 6150, Australia; 3 Biodiversity and Geosciences Program, Queensland Museum, South Brisbane, QL 4101, Australia

**Keywords:** biogeography, fellodistomidae, host-specificity, lecithostaphylinae, *Lintonium*, phylogeny

## Abstract

As part of a broad survey of the trematodes of damselfishes (Pomacentridae) in the tropical Indo-West Pacific, zoogonids were collected from multiple localities in Australia, New Caledonia, and French Polynesia. All zoogonid specimens collected were consistent with the subfamily Lecithostaphylinae, and morphological and molecular data (ITS2 and 28S rDNA, and *cox*1 mtDNA) were generated for most host-locality combinations to enable an integrative species delimitation. The collection comprised three species: *Deretrema stratiotes* n. sp. from four species of *Abudefduf* Forsskål from Ningaloo Reef in Western Australia, and two species consistent with the genus *Lecithostaphylus* Odhner, 1911 for which *Innuptacola* n. gen. is proposed based on phylogenetic and morphological distinction, the type-species *I. gibsoni* (Cribb, Bray & Barker, 1992) n. comb. (= *L. gibsoni*) from six species of *Abudefduf* in Ningaloo Reef, Queensland and New Caledonia, and *I. torquata* n. sp. from 12 pomacentrid species in Ningaloo Reef, the Great Barrier Reef in Queensland, and the Gambier Islands in French Polynesia. The new collection demonstrates that some zoogonid species are geographically widespread (from the Pacific Ocean to the Indian Ocean) and can infect a broad range of hosts (multiple genera within a family), whereas others are apparently geographically restricted and exhibit higher host-specificity (fishes within a single genus).

## Graphical abstract

**Figure:**
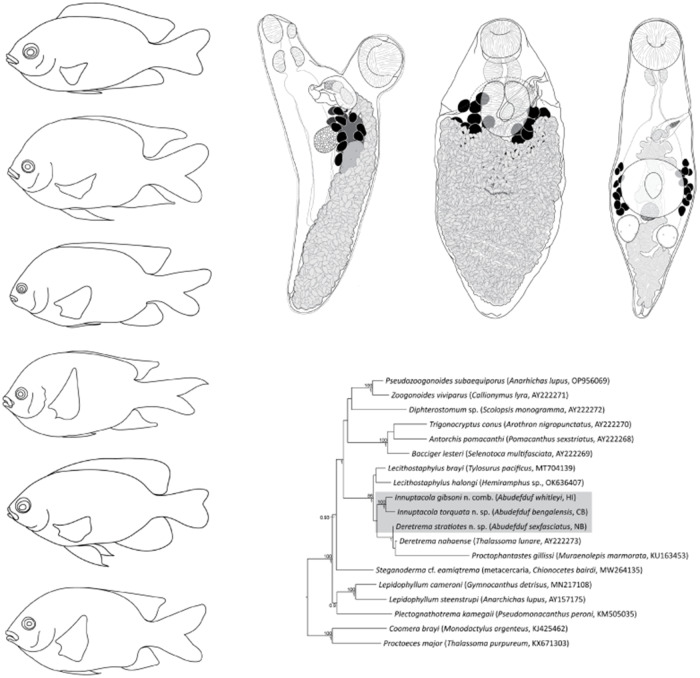

## Introduction

The Zoogonidae Odhner, 1902 is a family of microphalloid trematodes in which the hermaphroditic adults parasitize the gastrointestinal tract, the gall and urinary bladders, and the bile duct of mostly marine teleost fishes, and occasionally elasmobranchs and freshwater teleosts (Bray, 2008). Zoogonids are characterized by a spinous tegument, canalicular seminal receptacle, lateral genital pore and restricted fields of vitelline follicles. The family is currently divided into four subfamilies (Sokolov et al., 2021b): the Cephaloporinae Yamaguti, 1934 with three genera, the Lecithostaphylinae Odhner, 1911 with 19 genera, the Lepidophyllinae Stossich, 1904 with two genera, and the Zoogoninae Odhner, 1902 with 10 genera. This subfamilial division is consistent with the most recent molecular reconstruction of phylogeny for the Zoogonidae (Atopkin et al., 2022), with the exception of one nominal lecithostaphyline species *Steganoderma* cf. *eamiqtrema* Blend & Rácz, 2020, which did not form a clade with other represented lecithostaphyline taxa (Sokolov et al., 2021b).

The lecithostaphyline genera *Deretrema* Linton, 1910 and *Lecithostaphylus* Odhner, 1911 are the two richest zoogonid genera, comprising 16 and 13 species, respectively. Species of *Deretrema* infect the gall bladder of mostly perciform and some other eupercarian fishes, and species of *Lecithostaphylus* infect the intestine of primarily beloniform fishes. Morphologically, species of these two genera are distinguished by the distribution of the vitelline follicles and the protuberance of the ventral sucker. In species of *Deretrema*, the vitelline follicles typically extend into the forebody (between the ventral sucker and pharynx), and the ventral sucker is sessile, whereas in species of *Lecithostaphylus*, the vitelline follicles typically extend into the hindbody (often to the level of or beyond the testes) and the ventral sucker is often pedunculated.

Damselfishes (Pomacentridae) are among the richest and most abundant fishes on coral reefs (Parmentier and Frédérich, 2016). To date, there have been six zoogonids reported from pomacentrids (Table 1), including three species of *Deretrema* and two species of

**Table 1.** Records of pomacentrid-infecting zoogonids, including reports from non-pomacentrid hosts. Abbreviations: AU, Australia; AR, Argentina; PR, Puerto Rico; SC, the Seychelles; US, United States of America

Host species	Locality	References
***Deretrema fusillus*** Linton, 1910		
Pomacentridae: *Abudefduf saxatilis* (Linnaeus)	Dry Tortugas, Florida, US	Linton (1910); Manter (1934); Manter (1947)
	Media Luna Reef, La Parguera, PR	Dyer et al. (1985)
Exocoetidae: *Exocoetus volitans* Linnaeus	Woods Hole, Massachusetts, US	Linton (1940)
Haemulidae: *Haemulon album* Cuvier	Bermuda	Hanson (1950)
Haemulidae: *Haemulon macrostomum* Günther	Dry Tortugas, Florida, US	Linton (1910)
Labridae: *Decodon puellaris* (Poey)	Dry Tortugas, Florida, US	Manter (1934); Manter (1947)
Labridae: *Notolabrus parilus* (Richardson)	Point Peron, Western Australia, AU	Hall et al. (1999)
Lutjanidae: *Ocyurus chrysurus* (Bloch)	Dry Tortugas, Florida, US	Linton (1910); Manter (1947); Hanson (1950)
	Veracruz, Mexico	Montoya-Mendoza et al. (2014)
Mullidae: *Upeneus parvus* Poey	Dry Tortugas, Florida, US	Manter (1934)
Priacanthidae: *Priacanthus arenatus* Cuvier	Dry Tortugas, Florida, US	Manter (1947)
Serranidae: *Mycteroperca bonaci* (Poey)	Curaçao	Nahhas and Cable (1964)
Serranidae: *Serranus cabrilla* (Linnaeus)	Mediterranean Sea	Parukhin et al. (1971); Naidenova and Mordvinova (1997)
***Deretrema ludwicki*** **Toman, 1992**		
Pomacentridae: *Abudefduf septemfasciatus* (Cuvier)	Victoria, Mahé Island, SC	Toman (1992)
***Deretrema* sp.** **of Toman and Kamegai (1974)**		
Pomacentridae: *Abudefduf sordidus* (Forsskål)	Saipan, Northern Mariana Islands	Toman and Kamegai (1974)
***Diphterostomum americanum*** **Manter, 1947**		
Pomacentridae: *Stegastes leucostictus* (Müller & Troschel)	Biscayne Bay, Florida, US	Overstreet (1969)
Batrachoididae: *Opsanus beta* (Goode & Bean)	Snell Isle, Tampa Bay, Florida, US	Sogandares-Bernal and Hutton (1959); Hutton and Sogandares-Bernal (1960)
Gobiidae: *Gobiosoma robustum* Ginsburg	Mullet Key, Boca Ciega Bay, Florida, US	Sogandares-Bernal and Hutton (1959); Hutton and Sogandares-Bernal (1960)
Gobiidae: *Sufflogobius bibarbatus* (von Bonde)	Namibia	Gaevskaya and Aleshkina (1983)
Haemulidae: *Brachygenys chrysargyreum* (Günther)	Dry Tortugas, Florida, US	Manter (1947)
Lophiidae: *Lophius piscatorius* Linnaeus	Namibia	Gaevskaya and Aleshkina (1983)
Sparidae: *Archosargus rhomboidalis* (Linnaeus)	Biscayne Bay, Florida, US	Overstreet (1969)
Sparidae: *Lagodon rhomboides* (Linnaeus)	Bayboro Harbor, Tampa Bay, Florida, US	Sogandares-Bernal and Hutton (1959); Hutton and Sogandares-Bernal (1960)
	Biscayne, US	Overstreet (1969)
Sparidae: *Pagrus pagrus* (Linnaeus)	Quequén, AR	Schulze (1970)
Sparidae: *Sparus heterodus* Peters	South Atlantic Ocean	Parukhin (1966); Parukhin (1968); Parukhin (1976)
***Lecithostaphylus gibsoni*** **Cribb, Bray & Barker, 1992**		
Pomacentridae: *Abudefduf whitleyi* Allen & Robertson	Heron Island, Great Barrier Reef, AU	Cribb et al. (1992); Barker et al. (1994)
***Lecithostaphylus pomacentri*** **Toman, 1992**		
Pomacentridae: *Neopomacentrus taeniurus* (Bleeker)	St Anne Island, SC	Toman (1992)

*Lecithostaphylus*. Four of the six species are known only from pomacentrids: *Deretrema ludwicki* Toman, 1992 and *Lecithostaphylus pomacentri* Toman, 1992 from the Seychelles; *Lecithostaphylus gibsoni* Cribb, Bray & Barker, 1992 from the Great Barrier Reef; and *Deretrema* sp. from the Northern Mariana Islands in the western Pacific. The other two species, *Deretrema fusillus* Linton, 1910 and *Diphterostomum americanum* Manter, 1947, have each been reported from fishes spanning several families and mostly from the tropical northwestern Atlantic, although *Di. americanum* is known also from more temperate waters in the southern Atlantic off Argentina and Namibia, and *De. fusillus* has been reported from the Mediterranean and, perhaps doubtfully, Western Australia.

In the present study, specimens morphologically consistent with *Deretrema* and *Lecithostaphylus* were collected from 15 pomacentrid species from multiple localities across the Indo-West Pacific. The identity and phylogenetic relationships of these taxa are investigated here, with the description of a new genus and two new species.

## Materials and methods

Pomacentrid fishes were collected by barrier netting, line fishing or spear fishing at the following Indo-West Pacific localities: Amity Point (27°24′S, 153°26′E), North Stradbroke Island, and the Gold Coast Seaway (27°56′S, 153°25′E) in Moreton Bay, Heron Island (23°26′S, 151°54′E) and Lizard Island (14°40′S, 145°26′E) in the Great Barrier Reef, Queensland; Coral Bay (23°08′S, 113°46′E), North West Cape (21°50′S, 114°01′E), and Norwegian Bay (22°36′S, 113°40′E) in Ningaloo Reef, Western Australia; Nouméa (22°16′S, 166°26′E), New Caledonia; and the Gambier Islands (23°08′S, 134°57′W), French Polynesia. Fishes were examined for trematodes following the protocols of Cribb and Bray (2010). Trematodes collected were fixed in near-boiling saline and preserved in 80% ethanol for parallel morphological and molecular study. Some specimens were preserved in 10% formalin and were used only for morphological study. Where possible, trematode specimens were processed as either hologenophores or paragenophores (see Pleijel et al., 2008).

Specimens for morphological study were rinsed with distilled water, overstained in Mayer’s haematoxylin, destained in a 1% hydrochloric acid solution, neutralized in a 1% ammonium hydroxide solution, and dehydrated in a graded series of ethanol solutions. Specimens were then cleared in methyl salicylate and mounted on glass slides in Canada balsam. Morphometric data were taken using an Olympus SC50 camera mounted on a compound microscope (Olympus BX-53) with cellSens Standard imaging software. Drawings were made using a drawing tube attachment and digitized in Adobe Illustrator. Type- and voucher specimens are lodged at the Western Australian Museum (WAM) in Perth, and the Queensland Museum (QM) in Brisbane, Australia.

Genomic DNA was extracted using a standard phenol/chloroform extraction method (Sambrook and Russell, 2001). Sequence data were generated for the cytochrome *c* oxidase subunit 1 mitochondrial barcoding marker (*cox*1 mtDNA) region, and two ribosomal DNA (rDNA) regions, the partial D1–D3 fragment of the large ribosomal subunit RNA coding region (28S) and the entire second internal transcribed spacer region (ITS2) with short, flanking partial 5.8S and 28S (hereafter referred to as ‘ITS2’ for simplicity). These regions were amplified using the following primers: Dig_cox1Fa [5′-ATG ATW TTY TTY TTY YTD ATG CC-3′; Wee et al. (2017)] and Dig_cox1R [5′-TCN GGR TGH CCR AAR AAY CAA AA-3′; Wee et al. (2017)] for *cox*1, LSU5 [5′-TAG GTC GAC CCG CTG AAY TTA AGC-3′; Littlewood (1994)] and 1500 R [5′-GCT ATC CTG AGG GAA ACT TCG-3′; Snyder and Tkach (2001)] for 28S, and 3S [5′-GGT ACC GGT GGA TCA CGT GGC TAG TG-3′; Morgan and Blair (1995)] and ITS2.2 [5′-CCT GGT TAG TTT CTT TTC CTC CGC-3′; Cribb et al. (1998)] for ITS2. Polymerase chain reaction (PCR) for each region was performed using a TaKaRa PCR Thermal Cycler (see Cribb et al., 2022). Amplified DNA was sent to the Australian Genome Research Facility for purification and dual direction Sanger sequencing using the amplification primers for *cox*1 and ITS2, and the internal primers 300 F [5′-CAA GTA CCG TGA GGG AAA GTT-3′; Littlewood et al. (2000)] and ECD2 [5′-CTT GGT CCG TGT TTC AAG ACG GG-3′; Littlewood et al. (1997)] for the 28S region. Sequences were assembled and edited in Geneious Prime version 2021.11.0.9 (Kearse et al., 2012).

Genetic diversity and species boundaries were explored via separate alignments of the novel *cox*1 mtDNA and ITS2 rDNA sequences in MEGAX (Kumar et al., 2018) using MUSCLE (Edgar, 2004) with UPGMA clustering for iterations 1 and 2. To inform levels of differences in species delimitation, new *cox*1 data were generated for *Deretrema nahaense* Yamaguti, 1942 collected from two species of *Thalassoma* (Labridae) from the Great Barrier Reef in Queensland. The *cox*1 alignment was translated (echinoderm/flatworm mitochondrial code), examined for internal stop codons, and the correct reading frame was determined in Mesquite version 3.81 (Maddison and Maddison, 2023). All codon positions were then tested for non-stationarity in PAUP* version 4.0a (Swofford, 2003), and substitution saturation using the ‘Test of substitution saturation by Xia *et al*.’ function (Xia et al., 2003; Xia and Lemey, 2009) implemented in DAMBE version 7.2 (Xia, 2018); neither non-stationarity nor substitution saturation were detected, and thus, no codons were excluded from subsequent analyses. For the ITS2 alignment, ambiguously aligned base positions (bp) were few and were not masked or removed. The final *cox*1 and ITS2 datasets comprised 474 bp and 407 bp, respectively. Unrooted neighbour-joining analyses were conducted in MEGAX (Kumar et al., 2018) for each of the *cox*1 and ITS2 alignments with the following parameters: ‘test of phylogeny = bootstrap method’, ‘no. of bootstrap replications = 10 000’, ‘model/method = no. of differences’, ‘substitutions to include = d: transitions + transversions’, ‘rates among sites = uniform rates’, and ‘gaps/missing data treatment = pairwise deletion’. Pairwise differences for each alignment were estimated using the following parameters: ‘variance estimation method = none’, ‘model/method = no. of differences’, ‘substitutions to include = d: transitions + transversions’, ‘rates among sites = uniform rates’, and ‘gaps/missing data treatment = pairwise deletion’.

Taxa represented in the novel material were incorporated into broader phylogenetic reconstructions of the Zoogonidae based on the newly generated partial 28S rDNA sequences. These were aligned with comparable sequences for zoogonid taxa available in the GenBank database, together with comparable sequences for relevant taxa of the Faustulidae Poche, 1926 based on previous analyses by Cutmore et al. (2014) and Sokolov et al. (2021a), and two outgroup taxa from the Fellodistomidae Nicoll, 1909 (see Table 2). Sequences were aligned using MUSCLE version 3.7 through the CIPRES Portal (Miller et al., 2010) with UPGMA clustering for iterations 1 and 2. The alignment was refined (following Martin et al., 2018) in Mesquite Version 3.81 (Maddison and Maddison, 2023). Phylogenetic reconstructions were conducted using maximum likelihood and Bayesian inference analyses through the CIPRES Portal. The maximum likelihood analysis was run using RAxML Version 8.2.12 (Stamatakis, 2014) with 1000 bootstrap pseudoreplicates. The Bayesian inference analysis was run using MrBayes version 3.2.7a (Ronquist et al., 2012) with the following parameters: ‘ngen = 10 000 000’, ‘nruns = 2’, ‘nchains = 4’, ‘samplefreq = 1000’, ‘nst = 6’, ‘rates = gamma’, ‘ngammacat = 4’, ‘ratepr = fixed’, ‘sumt burnin value = 3000’, ‘sump burnin value = 3000’, and ‘burninfrac = 0.3’. Both analyses assumed the ‘GTR + I + Γ’ model of nucleotide substitution evolution; the best fitting models predicted via analysis in jModelTest Version 2.1.10 (Darriba et al., 2012) were ‘GTR + I + Γ’ and ‘TVM + Γ’ using the corrected Akaike Information Criterion and Bayesian Information Criterion, respectively.

**Table 2.** Information for trematode species used in the 28S rDNA analyses, including host species, locality and GenBank accession numbers. Abbreviations: AQ, Antarctica; AU, Australia; RU, Russia; UK, United Kingdom; VN, Vietnam

Trematode species	Host species	Locality	GenBank accession numbers	References
**Zoogonidae**				
**Subfamily Cephaloporinae**				
*Plectognathotrema kamegaii* Cutmore, Miller, Bray & Cribb, 2014	Monacanthidae: *Pseudomonacanthus peroni* (Hollard)	Ningaloo Reef, AU	KM505035	Cutmore et al. (2014)
**Subfamily Lecithostaphylinae**				
*Deretrema nahaense* Yamaguti, 1942	Labridae: *Thalassoma lunare* Linnaeus	Lizard Island, AU	AY222273	Olson et al. (2003)
*Lecithostaphylus brayi* Cabañas-Granillo, Solórzano-García, Mendoza-Garfias & Pérez-Ponce de León, 2020	Belonidae: *Tylosurus pacificus* (Steindachner)	Mexico	MT704139	Cabañas-Granillo et al. (2020)
*Lecithostaphylus halongi* Atopkin, Besprozvannykh, Ha, Nguyen & Nguyen, 2022	Hemiramphidae: *Hemiramphus* sp.	Ha Long Bay, VN	OK636407	Atopkin et al. (2022)
*Proctophantastes gillissi* (Overstreet & Pritchard, 1977) Bray & Gibson, 1986	Muraenolepididae: *Muraenolepis marmorata* Günther	Ross Sea, AQ	KU163453	Sokolov et al. (2016)
*Steganoderma* cf. *eamiqtrema* Blend & Rácz, 2020	Oregoniidae: *Chionoecetes bairdi* Rathbun	Sea of Okhotsk, RU	MW264135	Sokolov *et al*. (2021b)
**Subfamily Lepidophyllinae**				
*Lepidophyllum cameroni* Arai, 1969	Cottidae: *Gymnocanthus detrisus* Gilbert & Burke	Kamchatka Krai, RU	MN217108	Sogrina *et al*. (unpub)
*Lepidophyllum steenstrupi* Odhner, 1902	Anarhichadidae: *Anarhichas lupus* Linnaeus	North Sea, UK	AY157175	Lockyer et al. (2003)
**Subfamily Zoogoninae**				
*Diphterostomum* sp.	Nemipteridae: *Scolopsis monogramma* (Cuvier)	Heron Island, AU	AY222272	Olson et al. (2003)
*Pseudozoogonoides subaequiporus* (Odhner, 1911) Bray & Gibson, 1986	Anarhichadidae: *Anarhichas lupus*	Keret Archipelago, RU	OP956069	Kremnev et al. (2023)
*Pseudozoogonoides ugui* Shimazu, 1974	Cyprinidae: *Pseudaspius hakonensis* (Günther)	Primorsky Region, RU	PP317550	Atopkin et al. (2024)
*Zoogonoides viviparus* (Olsson, 1868) Odhner, 1902	Callionymidae: *Callionymus lyra* Linnaeus	North Sea, UK	AY222271	Olson et al. (2003)
**Faustulidae**				
*Antorchis pomacanthi* (Hafeezullah & Siddiqi, 1970) Machida, 1975	Pomacanthidae: *Pomacanthus sexstriatus* (Cuvier)	Heron Island, AU	AY222268	Olson et al. (2003)
*Bacciger lesteri* Bray, 1982	Scatophagidae: *Selenotoca multifasciata* (Richardson)	Moreton Bay, AU	AY222269	Olson et al. (2003)
*Trigonocryptus conus* Martin, 1958	Tetraodontidae: *Arothron nigropunctatus* (Bloch & Schneider)	Heron Island, AU	AY222270	Olson et al. (2003)
**Fellodistomidae (Outgroup)**				
*Coomera brayi* Dove & Cribb, 1995	Monodactylidae: *Monodactylus argenteus* (Linnaeus)	Moreton Bay, AU	KJ425462	Cribb et al. (2014)
*Proctoeces major* Yamaguti, 1934	Labridae: *Thalassoma purpureum* (Forsskål)	Moreton Bay, AU	KX671303	Wee et al. (2017)

To assist with future systematic studies, individual sequences of each unique host-parasite-locality combination represented in each of the datasets (*cox*1 mtDNA, and ITS2 and 28S rDNA) were uploaded to GenBank; relevant accession numbers are provided within the description of each taxa.

## Results

### General results

A total of 1,595 fishes belonging to 60 pomacentrid species were examined for trematodes. The only zoogonids encountered from these dissections were specimens consistent with current diagnosis of *Lecithostaphylus* or *Deretrema*, and were from 15 pomacentrid species belonging to the following genera: *Abudefduf* Forsskål, *Acanthochromis* Gill, *Neoglyphidodon* Allen, *Plectroglyphidodon* Fowler & Ball, and *Pomacentrus* Lacépède.

### Molecular and phylogenetic results

**Figure 1.**
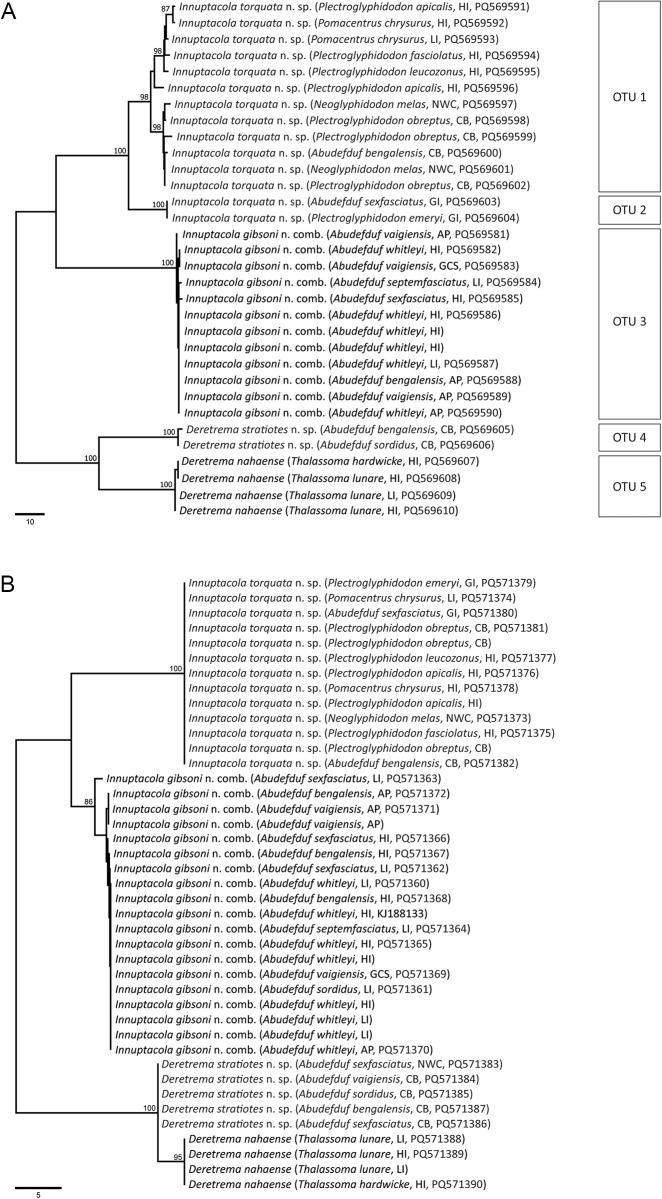
Phylograms from unrooted neighbour-joining analyses of the (A) *cox*1 mtDNA and (B) ITS2 rDNA datasets. Bootstrap support values (>85) are shown at the nodes. Scale bars indicate the number of base differences. Abbreviations: AP, Amity Point; CB, Coral Bay; GCS, Gold Coast Seaway; GI, Gambier Islands; HI, Heron Island; LI, Lizard Island; NB, Norwegian Bay; NWC, North West Cape.

*cox*1 mtDNA data were generated for 32 zoogonid specimens (comprising 26 consistent with the diagnosis of *Lecithostaphylus* and six consistent with *Deretrema*), representing 23 host-parasite-locality combinations. The unrooted neighbour-joining analysis of the *cox*1 sequence dataset (Figure 1A) resolved five operational taxonomic units (OTUs), well-supported clades with less than 20 bp intra-clade variation (Table 3). OTUs 1–3 relate to specimens consistent with *Lecithostaphylus*: OTU 1 comprises sequences for specimens from the intestine of eight species of pomacentrids from the Great Barrier Reef and Ningaloo Reef; OTU 2 comprises two sequences for specimens from the intestine of two species of pomacentrids from the Gambier Islands; and OTU 3 comprises sequences for specimens from the lower intestine of six species of *Abudefduf* from the Great Barrier Reef and Moreton Bay. OTUs 4 and 5 relate to specimens consistent with *Deretrema*: OTU 4 comprises two sequences for samples from the gall bladder of two species of *Abudefduf* from Ningaloo Reef; and OTU 5 comprises sequences for samples from the gall bladder of two labrid species from the Great Barrier Reef. OTUs 2–5 have very low intra-clade variation, at up to six bp, whereas OTU 1 has significant intra-clade variation and some geographic structuring. Within OTU 1, specimens from the Great Barrier Reef formed a poorly-supported clade, varying at up to 12 bp, sister to a clade of samples from Ningaloo Reef; these two clades differed at 10–18 bp.

**Table 3.** Pairwise base position differences between operational taxonomic units (OTU) for the cox1 mtDNA sequence dataset

	OTU 1	OTU 2	OTU 3	OTU 4	OTU 5
OTU 1	0–18				
OTU 2	24–34	0			
OTU 3	82–87	84–86	0–2		
OTU 4	115–120	111–113	117–120	1	
OTU 5	113–121	111–112	117–120	56–58	0–1

ITS2 rDNA data were generated for 39 zoogonid specimens (31 specimens consistent with *Lecithostaphylus* and eight specimens consistent with *Deretrema*), representing 29 host-parasite-locality combinations. The unrooted neighbour-joining analysis of the ITS2 sequence dataset resolved four well-supported clades (Figure 1B): two comprising specimens consistent with *Lecithostaphylus* and two comprising specimens consistent with *Deretrema*. Eighteen ITS2 sequences relating to *cox*1 OTU 3 (from 11 host-locality combinations comprising multiple species of *Abudefduf* in Queensland) were almost identical with variation at up to three bp; these data were identical to the existing GenBank sequence of *Lecithostaphylus gibsoni* [GenBank accession number KJ188133, as *Steganoderma gibsoni* from *Abudefduf whitleyi* Allen & Robertson (see Barnett et al., 2014)]. Thirteen ITS2 sequences relating to *cox*1 OTUs 1 and 2, representing 10 host-locality combinations spanning infections from pomacentrids from four genera from Ningaloo Reef, the Great Barrier Reef and the Gambier Islands, were identical and these sequences differ from those of the other *Lecithostaphylus* clade (i.e. OTU 3 = *L. gibsoni*) at 18–20 bp. Three ITS2 sequences were generated for *cox*1 OTU 5, all from labrids from the Great Barrier Reef; these sequences were identical and matched the existing GenBank sequence of *Deretrema nahaense* [GenBank accession number KJ188135, from *Thalassoma lunare* (Linnaeus) at Heron Island (see Barnett et al., 2014)]. Five ITS2 sequences were generated for *cox*1 OTU 4, all from species of *Abudefduf* from Ningaloo Reef; these sequences were identical and differed from those of *D. nahaense* (OTU 5) at three bp.

**Figure 2.**
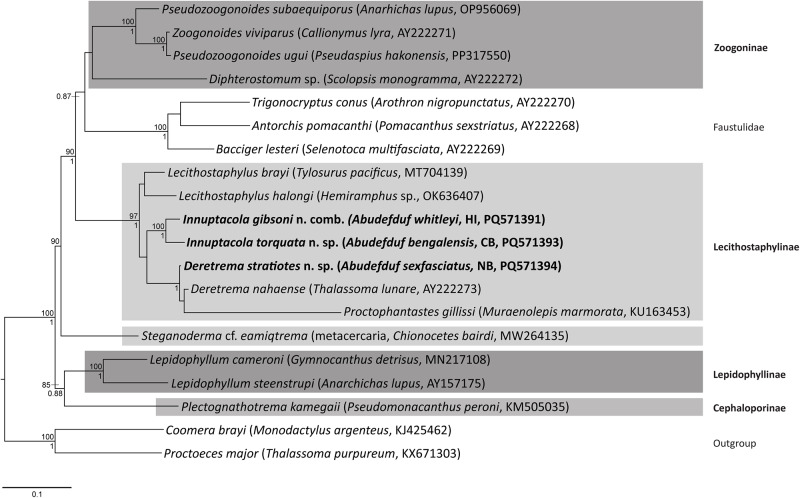
Relationships of the Zoogonidae (highlighted in shaded boxes) and the Faustulidae based on the maximum likelihood analysis of the 28S dataset. Bootstrap support values (>85) are shown above the node and posterior probabilities (>0.85) are shown below the node. Newly generated sequences are indicated in bold. The scale bar indicates the number of substitutions per sites. Abbreviations: CB, Coral Bay; HI, Heron Island; NB, Norwegian Bay.

Maximum likelihood and Bayesian inference analyses of the 28S rDNA dataset produced phylograms with identical topologies, and included representatives from all four zoogonid subfamilies (Figure 2). The novel sequences all resolved within the *Lecithostaphylinae sensu stricto* clade. Notably, OTU 4, consistent with *Deretrema*, formed a clade with *D. nahaense* (OTU 5) + *Proctophantastes gillissi* (Overstreet & Pritchard, 1977) Bray & Gibson, 1986; *P. gillissi* has a branch length in this clade that is longer than those separating the zoogonid subfamilies, suggesting that it is likely distinct from the two *Deretrema* species despite the resolved topology. Sequences of specimens from pomacentrids consistent with *Lecithostaphylus* formed a strongly supported clade resolving as sister to the clade of *Deretrema* + *Proctophantastes*. This clade then was sister to a clade of two species of *Lecithostaphylus* from beloniform fishes, *L. brayi* Cabañas-Granillo, Solórzano-García, Mendoza-Garfias & Pérez-Ponce de León, 2020 + *L. halongi* Atopkin, Besprozvannykh, Ha, Nguyen & Nguyen, 2022. The lecithostaphyline clade resolved sister to a moderately to strongly supported clade of four zoogonine species, *Pseudozoogonoides subaequiporus* (Odhner, 1911) Bray & Gibson, 1986 and *P. ugui* Shimazu, 1974 + *Zoogonoides viviparus* (Olsson, 1868), and a *Diphterostomum* sp., and three species belonging to the ‘microphalloid’ clade of the Faustulidae *sensu lato, Antorchis pomacanthi* (Hafeezullah & Siddiqi, 1970) Machida, 1975 + *Trigonocryptus conus* Martin, 1958, and *Bacciger lesteri* Bray, 1982. This lecithostaphyline and zoogonine + faustulid clade is sister to a sequence generated from a metacercarial sample and identified as *Steganoderma* cf. *eamiqtrema* Blend & Rácz, 2020, which also belongs to the Lecithostaphylinae. The two remaining zoogonid subfamilies are represented by *Lepidophyllum cameroni* Arai, 1969 + *L. steenstrupi* Odhner, 1902 (Lepidophyllinae), which forms a well-supported clade with *Plectognathotrema kamegaii* Cutmore, Miller, Bray & Cribb, 2014 (Cephaloporinae), and is sister to the lecithostaphyline, zoogonine and faustulid clade.

### Synthesis

Following the species recognition criteria proposed by Bray et al. (2022), an integrative approach combining morphological, molecular and host data was used in this study. On this basis, the new collection of zoogonids from Indo-West Pacific pomacentrids comprise three species from two genera. All specimens from pomacentrids consistent with *Deretrema* (*cox*1 OTU 4) represent a single undescribed species; this new species is formally described based on specimens from four species of *Abudefduf* Forsskål from Ningaloo Reef in Western Australia. The specimens consistent with *Lecithostaphylus* (*cox*1 OTUs 1–3) are interpreted as representing two species. *cox*1 OTU 3 is identified as a known species (*L. gibsoni*) from six species of *Abudefduf* at six localities. *cox*1 OTUs 1 and 2 are interpreted as a single species new to science, which is described based on material from 12 pomacentrid species from five localities. Based on distinctions in phylogenetic topology, morphology and host affinity, these two species are clearly distinct from other known species of *Lecithostaphylus*, and thus a new genus is proposed to accommodate them.

### Taxonomy

Family **Zoogonidae** Odhner, 1902

Subfamily **Lecithostaphylinae** Odhner, 1911

#### *Innuptacola* n. gen

**Diagnosis**: Body small, pyriform when viewed dorsoventrally (or triangular when viewed laterally). Tegument spinous. Oral sucker globular, opening subterminally. Ventral sucker globular, equal to or larger than oral sucker, pedunculate but may be withdrawn, in mid-anterior half of body. Prepharynx short. Pharynx rounded, smaller than oral and ventral suckers. Oesophagus short. Intestinal bifurcation in mid-anterior half of body. Caeca extend into hindbody, terminate blindly in posterior third of body. Testis subglobular, separate, in mid-body. Cirrus-sac claviform, can be elongated, overlaps ventral sucker. Seminal vesicle bipartite. Pars prostatica vesicular, shorter than seminal vesicle. Ejaculatory duct short. Genital atrium small. Genital pore sinistral, opens at level of mid-forebody or ventral sucker. Ovary globular, median, in anterior hindbody, at level of or anterior to testes, may overlap ventral sucker dorsally. Canalicular seminal receptacle reniform or oval, median, inter-testicular. Uterus in hindbody. Eggs numerous, operculate, tanned. Vitelline follicles irregularly globular, contiguous or slightly separated, clustered in separate fields either side of ventral sucker, may extend into anterior hindbody. Excretory vesicle I-shaped/saccular. Excretory pore dorsally sub-terminal or terminal. In intestine of pomacentrid fishes.

Type-species: *Innuptacola gibsoni* (Cribb, Bray & Barker, 1992) n. comb. (= *Lecithostaphylus gibsoni* Cribb , Bray & Barker, 1992).

Other species: *Innuptacola torquata* n. sp.

ZooBank Life Science Identifier: urn:lsid:zoobank.org:act:979584AD-7A05-497D-8311-373AC52B0E11.

Etymology: The epithet ‘*Innuptacola*’ means inhabitant of damsels, referring to the included species which are, thus far, only found in pomacentrids, the damselfishes. ‘*Innuptacola’* is a combination of the Latin adjective *innŭ*pta, meaning unmarried (i.e. a damsel) and noun *-cola*, meaning inhabitant.

**Remarks**: Morphological and phylogenetic analyses clearly place *L. gibsoni* and the new species of *Lecithostaphylus* in the subfamily Lecithostaphylinae. However, the paraphyly of *Lecithostaphylus* in the 28S rDNA analyses, combined with the distinct host distribution and morphological differences, suggest that these species are best considered in a distinct genus. A detailed case for the proposal of *Innuptacola* n. gen. is presented in the Discussion.

#### *Innuptacola gibsoni* (Cribb, Bray & Barker, 1992) n. comb (Figure 3)

Synonyms: *Lecithostaphylus gibsoni* Cribb, Bray & Barker, 1992; *Steganoderma gibsoni* Cribb, Bray & Barker, 1992.

**Figure 3.**
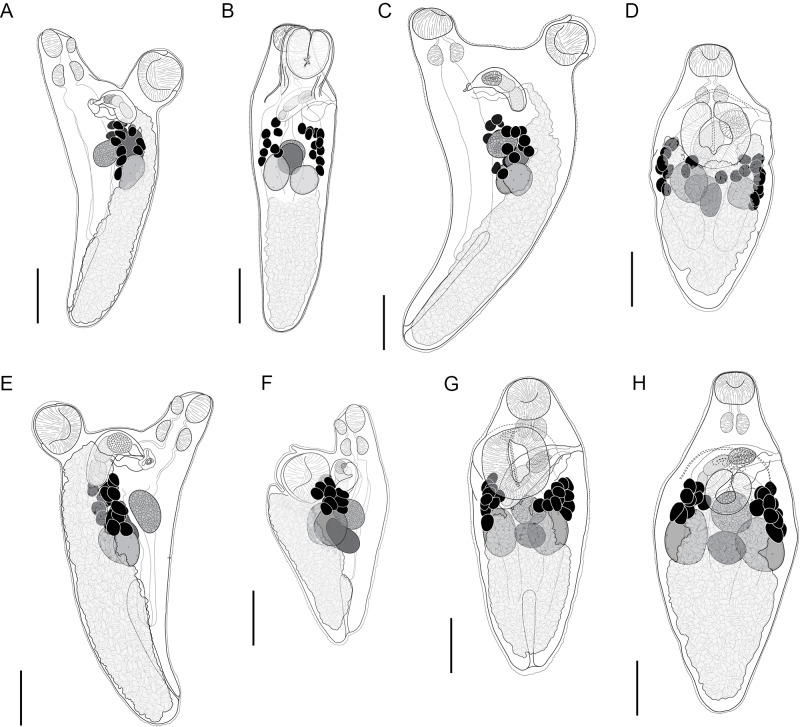
*Innuptacola gibsoni* (Cribb, Bray & Barker, 1992) n. comb.: (A, B) ex *Abudefduf whitleyi*, Heron Island, Great Barrier Reef; (C, D) ex *A. whitleyi*, Lizard Island, Great Barrier Reef; (E) ex *A. vaigiensis*, Moreton Bay; (F, G) ex *A. bengalensis*, Ningaloo Reef; (H) ex *A. whitleyi*, New Caledonia. (A, C, E, F) Lateral view; (B, D, G, H) Dorso-ventral view. Scale bars = 200 *μ*m.

Type-host: *Abudefduf whitleyi* Allen & Robertson, Whitley’s sergeant (Pomacentridae).

Type-locality: Heron Island, southern Great Barrier Reef, Queensland, Australia.

Other records: Barker et al. (1994); Barnett et al. (2014).

***New material***

New hosts: *Abudefduf bengalensis* (Bloch), Bengal sergeant; *Abudefduf septemfasciatus* (Cuvier), Banded sergeant; *Abudefduf sexfasciatus* (Lacépède), Scissortail sergeant; *Abudefduf sordidus* (Forsskål), Blackspot sergeant; *Abudefduf vaigiensis* (Quoy & Gaimard), Indo-Pacific sergeant (Pomacentridae).

Known host: *A. whitleyi*.

New localities: Lizard Island (14°40′S, 145°26′E), northern Great Barrier Reef, Amity Point (27°24′S, 153°26′E) and the Gold Coast Seaway (27°56′S, 153°25′E), Moreton Bay, Queensland, Australia; Coral Bay (23°08′S, 113°46′E), North West Cape (21°50′S, 114°01′E), and Norwegian Bay (22°36′S, 113°40′E), Ningaloo Reef, Western Australia, Australia; Nouméa (22°16′S, 166°26′E), New Caledonia.

Known locality: Heron Island.

Abundance and prevalence: Heron Island: one specimen from one of 50 (2%) *A. bengalensis*; one specimen from one of 30 (3%) *A. sexfasciatus*; 51 specimens from 24 of 60 (40%) *A. whitleyi*. Lizard Island: two specimens from two of 13 (15%) *A. bengalensis*; eight specimens from three of five (60%) *A. septemfasciatus*; seven specimens from seven of 23 (30%) *A. sexfasciatus*; one specimen from one of three (33%) *A. sordidus*; 10 specimens from four of 15 (27%) *A. whitleyi*. Amity Point: two specimens from two of 65 (3%) *A. bengalensis*; three specimens from three of 25 (14%) *A. vaigiensis*; two specimens from two of 29 (7%) *A. whitleyi*. Gold Coast Seaway: one specimen from one of four (25%) *A. vaigiensis*. Coral Bay: two specimens from one of two (50%) *A. sordidus*. North West Cape: one specimen from one of 11 (10%) *A. bengalensis*. Norwegian Bay: five specimens from one of one (100%) *A. bengalensis*. Nouméa: two specimens from two of three (67%) *A. sexfasciatus*; 20 specimens from seven of seven (100%) *A. whitleyi*.

Site in host: Lower intestine.

Deposited material: 44 vouchers (QM G241494–241520, G241534–241543; WAM V 12847–12850).

Representative DNA sequences: ITS2 rDNA, 18 sequences (13 submitted to GenBank, PQ571360–PQ571372); partial 28S rDNA, two sequences (both submitted to GenBank, PQ571391–PQ571392); partial *cox*1 mtDNA, 12 sequences (10 submitted to GenBank, PQ569581–PQ569590).

**Description**: [Based on 25 whole mounted specimens and 10 hologenophores. See Table 4 for measurements.] Body broadly oval/pyriform, widest at level of ventral sucker. Tegument covered in fine spines, most dense in forebody. Oral sucker globular. Ventral sucker oval, pedunculate (retracted in some specimens, most clearly visible in laterally mounted specimens) with longitudinal aperture. Prepharynx observed occasionally in dorso-ventrally mounted specimens, clearly visible in laterally mounted specimens. Pharynx round. Oesophagus short. Intestine bifurcates at level of ventral sucker. Caeca extend posteriorly into hindbody, terminate blindly in posterior third of body. Testes subglobular, separate, opposite, overlap caeca, slightly overlap posterior regions of ovary and seminal receptacle. Cirrus-sac elongated oblong, thick-walled, overlaps ventral sucker dextrally. Internal seminal vesicle bipartite, occupies two thirds of cirrus-sac. Pars prostatica vesicular, occupies anterior third of cirrus-sac. Ejaculatory duct short. Genital pore sinistral, submarginal, mid-way between oral and ventral suckers. Ovary subglobular, median, anterior to testes, overlaps seminal receptacle dorsally. Canalicular seminal receptacle reniform, median. Egg-forming complex not observed. Vitelline follicles in two fields, clustered laterally in anterior hindbody, pre-testicular, predominantly extracaecal, irregularly globular, contiguous or slightly separated; vitelline fields with 9–12 follicles. Uterus occupies entire hindbody. Metraterm dorsal to ventral sucker and parallels cirrus-sac to genital pore. Eggs numerous, tanned, operculate. Excretory vesicle I-shaped, extends to level of caecal ends. Excretory pore terminal.

**Table 4.** Measurements of *Innuptacola gibsoni* (Cribb, Bray & Barker, 1992) n. comb. from five indo-west pacific localities. Abbreviations: BE, body extremity; BL, body length; EV, excretory vesicle; FBL, forebody length; GP, genital pore; L, length; OS, oral sucker; PP, pars prostatica; SR, seminal receptacle; SV, seminal vesicle; VF, vitelline follicle; VS, ventral sucker; W, width.

	Heron Island	Lizard Island	Moreton Bay	Ningaloo Reef	New Caledonia
*n*	7	9	4	5	5
Body L	985–1494 (1244)	743–1220 (1034)	975–1193 (1084)	914–1158 (1075)	1016–1233 (1123)
Body W	344–398 (371)	240–561 (401)	357–473 (414)	451–477 (462)	338–512 (406)
Forebody L	176–326 (260)	194–299 (228)	218–340 (298)	170–246 (210)	214–353 (277)
FBL % BL		21.6–26.1 (24.1)	22–27 (25)	15.7–21.8 (19.6)	18.9–28.6 (24.7)
Hindbody L	745–1050 (886)	448–976 (630)	629–820 (725)	535–635 (605)	556–892 (703)
Ant. BE to GP	278–315 (296)	230–349 (300)	283–358 (309)	229–281 (260)	239–282 (259)
Post-caeca L	267–385 (309)	177–332 (259)	212–343 (278)	231–307 (262)	273–287 (280)
Post-testes L	374–634 (496)	297–555 (424)	328–580 (454)	372–485 (431)	444–544 (504)
Post-ovary L	491–687 (593)	567	661	437–549 (512)	463–644 (570)
OS L	99–137 (114)	66–124 (102)	99–125 (113)	100–147 (128)	102–112 (109)
OS W	99–163 (130)	81–174 (130)	130–158 (140)	116–168 (151)	127–144 (136)
Pre-pharynx L	15–31 (22)	21–29 (24)	18–35 (24)	18–28 (23)	23–34 (29)
Pharynx L	73–115 (94)	53–87 (74)	89–98 (93)	66–90 (79)	65–76 (70)
Pharynx W	105–145 (127)	77–138 (119)	120–149 (130)	103–127 (113)	98–122 (110)
Oesophagus	117	61		62–107 (83)	62–69 (66)
VS L	173–211 (190)	128–205 (162)	166–195 (178)	197–261 (240)	166–211 (184)
VS L/OS L	1.5–1.8 (1.7)	1.4–1.9 (1.6)	1.4–1.7 (1.6)	1.7–2.2 (1.9)	1.5–1.9 (1.7)
VS W	157–205 (178)	103–172 (147)	158–181 (170)	186–251 (228)	163–203 (184)
VS W/OS W	1.1–1.6 (1.4)	1–1.3 (1.1)	1.1–1.3 (1.2)	1.4–1.6 (1.5)	1.2–1.5 (1.4)
PP L	67–102 (82)	61–130 (84)	54–94 (71)	64–122 (94)	74–113 (98)
PP W	52–71 (63)	37–73 (55)	54–74 (61)	64–80 (73)	58–74 (67)
Ant. SV L	79–108 (94)	65–115 (85)	72–105 (93)	60–123 (94)	87–97 (91)
Ant. SV W	31–72 (48)	31–59 (45)	30–72 (52)	44–65 (55)	53–62 (57)
Post. SV L	40–74 (58)	42–87 (63)	75–81 (78)	31–74 (53)	83–87 (85)
Post. SV W	37–54 (43)	35–53 (42)	43–65 (54)	37–45 (41)	55–78 (66)
Ovary L	49–97 (79)	52–98 (70)	72	93–111 (103)	111–136 (120)
Ovary W	103–195 (140)	84–155 (120)	152	107–131 (121)	109–147 (120)
SR L	56–138 (92)	69–110 (89)	99	59–101 (87)	59–175 (115)
SR W	94–163 (132)	96–149 (122)	119–159 (139)	84–159 (135)	87–116 (121)
Right testis L	125–239 (168)	96–147 (123)	140	171–183 (179)	163–221 (189)
Right testis W	76–143 (106)	84–122 (97)	97	116–132 (123)	140–155 (147)
Left testis L	127–201 (152)	99–134 (112)	126	142–168 (159)	148–194 (170)
Left testis W	68–127 (95)	75–124 (95)	81	104–143 (125)	120–175 (142)
Egg L	33–39 (35)	33–39 (35)	31–37 (33)	30–36 (33)	34–39 (37)
Egg W	15–19 (17)	15–19 (17)	14–20 (18)	14–17 (16)	14–18 (17)
VF L	35–78 (52)	32–55 (44)	48–59 (52)	34–47 (43)	41–57 (52)
VF W	28–60 (40)	31–42 (38)	41–49 (43)	38–53 (47)	37–54 (45)
EV L	263–382 (331)	196–473 (358)	288–463 (393)	233–314 (274)	269–394 (328)

**Remarks**: New specimens of *I. gibsoni* n. comb. were collected from six *Abudefduf* spp. from multiple localities across the Central Indo-Pacific, including from the type-host-locality combination. Genetic data were generated for specimens collected from off Heron Island, Lizard Island, and Moreton Bay (collectively referred to as the Queensland coast); it was not possible to generate genetic data for specimens from New Caledonia and Ningaloo Reef.

**Figure 4.**
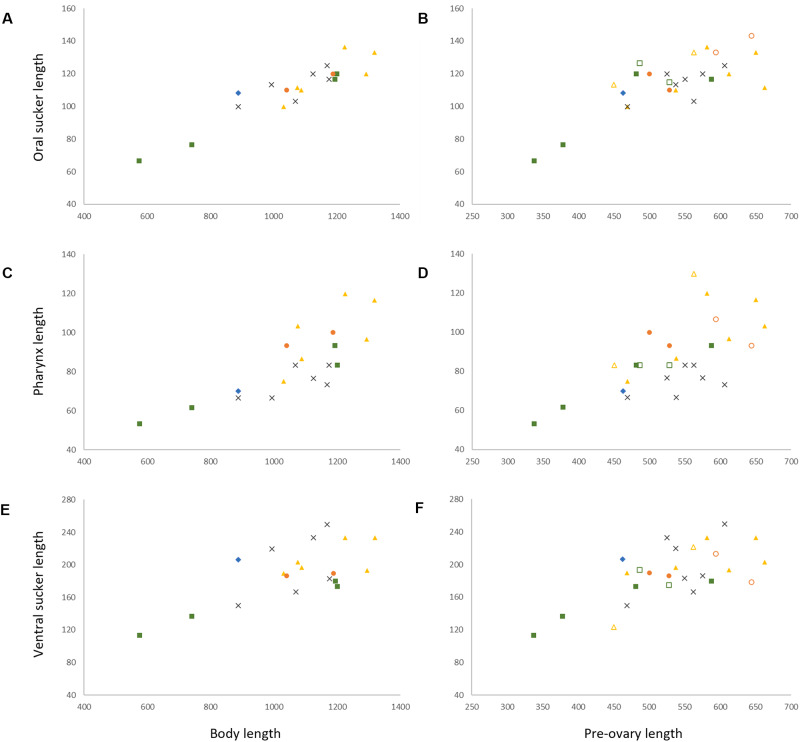
Morphometric comparisons of laterally mounted specimens of *Innuptacola gibsoni* (Cribb, Bray & Barker, 1992) n. comb. collected from species of *Abudefduf* from Heron Island, Lizard Island, Moreton Bay, Ningaloo Reef, and New Caledonia. Each scatter plot represents body length vs: (A) oral sucker length, (C) pharynx length and (E) ventral sucker, and pre-ovary length vs: (B) oral sucker length, (D) pharynx length and (F) ventral sucker length. Heron Island: yellow, △, hologenophore, ▲, whole; Lizard Island: green, □, hologenophore, ▪, whole; Moreton Bay: orange, ○, hologenophore, ●, whole; New Caledonia: grey, ×, whole; Ningaloo Reef: blue, ◆, whole.

Specimens of *I. gibsoni* exhibit variation in the shape and size of the body, cirrus-sac and suckers. The body shape varies from being slightly oblong to ovate, with further variation in the hindbody where the posterior extremity tapers either gradually or sharply to a blunt point. The ovate shape and sharp tapering is especially noticeable in specimens from off Lizard Island, Ningaloo Reef and New Caledonia. In some specimens from off Lizard Island, the seminal vesicle and pars prostatica are roughly as large as the testes and ovary, whereas in specimens from other localities, these organs are typically smaller. Plots of the maximum sucker and pharynx lengths relative to body length and pre-ovary length (to allow the inclusion of hologenophores) show that specimens from each locality overlap with no distinct patterns that would indicate the presence of multiple species (Figure 4). The morphological variation seen in the present collection of *I. gibsoni* does not appear to be related to either host or geographic distribution. However, some of the variation (particularly the body shape) may be attributed to the mounting process whereby specimens may have been slightly distorted by the slide cover or the positioning of the specimen (i.e. dorso-ventrally *vs* laterally mounted specimens).

The lack of genetic data limits our capacity to definitively identify the specimens from New Caledonia and Ningaloo Reef as *I. gibsoni*, especially since the present collection exhibits significant morphological variation. However, the specimens from these localities were collected from the same hosts as material from Queensland (*A. sexfasciatus* and *A. whitleyi* from New Caledonia, and *A. bengalensis* and *A. sordidus* from Ningaloo Reef), and the morphometric ranges were generally within or overlapping the ranges of specimens from the Queensland coast. Furthermore, given that the distribution of the second species of *Innuptacola* (described below) extends from Ningaloo Reef (Indian Ocean) through to the Great Barrier Reef and French Polynesia (Pacific Ocean), there is no reason to doubt that *I. gibsoni* extends to New Caledonia or Ningaloo Reef.

**Figure 5.**
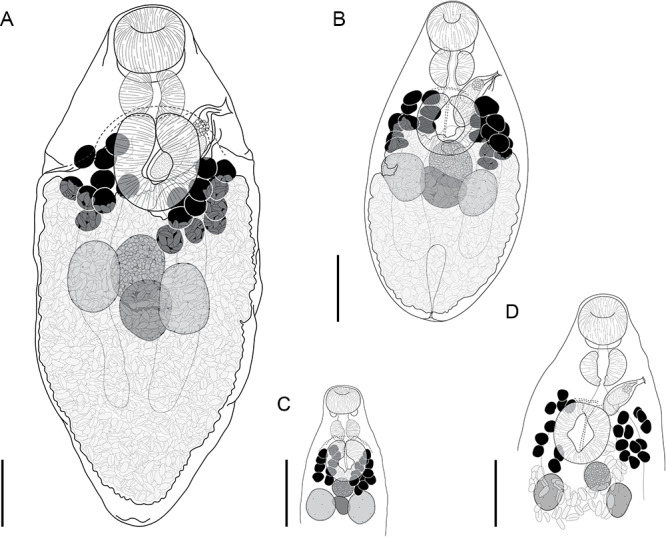
*Innuptacola torquata* n. sp.: (A) Holotype ex *Plectroglyphidodon obreptus*, Coral Bay, Ningaloo Reef; (B) Paragenophore ex *Plectroglyphidodon emeryi*, the Gambier Islands, French Polynesia; (C) Hologenophore ex *Pomacentrus chrysurus*, Heron Island, Great Barrier Reef; (D) Hologenophore ex *Pomacentrus chrysurus*, Lizard Island, Great Barrier Reef. Scale bars = 200 *μ*m.

#### *Innuptacola torquata* n. sp. (Figure 5)

Type-host: *Plectroglyphidodon obreptus* (Whitley), Western gregory (Pomacentridae).

Type-locality: Coral Bay (23°08′S, 113°46′E), Ningaloo Reef, Western Australia, Australia.

Other hosts: *Abudefduf bengalensis* (Bloch), Bengal sergeant; *Abudefduf septemfasciatus* (Cuvier), Banded sergeant; *Abudefduf sexfasciatus* (Lacépède), Scissortail sergeant; *Acanthochromis polyacanthus* (Bleeker), Spiny chromis; *Neoglyphidodon melas* (Cuvier), Black damsel; *Plectroglyphidodon apicalis* (De Vis), Australian gregory; *Plectroglyphidodon emeryi* (Allen & Randall), Emery’s gregory; *Plectroglyphidodon fasciolatus* (Ogilby), Pacific gregory; *Plectroglyphidodon leucozonus* (Bleeker), Whiteband damsel; *Pomacentrus amboinensis* Bleeker, Ambon damsel; *Pomacentrus chrysurus* Cuvier, Whitetail damsel (Pomacentridae).

Other localities: Gambier Islands (23°08′S, 134°57′W), French Polynesia; Heron Island (23°26′S, 151°54′E), southern Great Barrier Reef, and Lizard Island (14°40′S, 145°26′E), northern Great Barrier Reef, Queensland, Australia; North West Cape (21°50′S, 114°01′E), Ningaloo Reef, Western Australia, Australia.

Abundance and prevalence: Coral Bay: one specimen from one of two (50%) *Abu. bengalensis*; 13 specimens from six of seven (86%) *Ple. obreptus*. Gambier Islands: one specimen from one of three (33%) *Abu. septemfasciatus*; one specimen from one of four (25%) *Abu. sexfasciatus*; six specimens from three of six (50%) *Ple. emeryi*. Heron Island: one specimen from one of 78 (1%) *Aca. polyacanthus*; two specimens from two of 77 (2%) *Ple. apicalis*; one specimen from one of five (20%) *Ple. fasciolatus*; one specimen from one of five (20%) *Ple. leucozonus*; one specimen from one of 32 (3%) *Pom. chrysurus*. Lizard Island: one specimen from one of 43 (2%) *Pom. amboinensis*; two specimens from two of 32 (6%) *Pom. chrysurus*. North West Cape: six specimens from one of one (100%) *N. melas*.

Site in host: Intestine.

Deposited material: Holotype (WAM V 12836) and 20 paratypes (WAM V 12837–12846; QM G241521–241526, G241544–241547).

Representative DNA sequences: ITS2 rDNA, 13 sequences (10 submitted to GenBank, PQ571373–PQ571382); partial 28S rDNA, one sequence (submitted to GenBank, PQ571393); partial *cox*1 mtDNA, 14 sequences (all submitted to GenBank, PQ569591–PQ569604).

ZooBank Life Science Identifier: urn:lsid:zoobank.org:act:DD1B3F80-3C9D-4465-8E06-6FCE13BD1678.

Etymology: The epithet ‘*torquata*’, based on the Latin adjective *torquāta* for collared, refers to distinct post-oral collar in this species.

**Description**: [Based on 19 whole mounted specimens and 17 hologenophores.] Body oval to pyriform, widest at level of testes, 584–1329 × 250–617 (1083 × 450). Tegument covered in fine spines, most dense anteriorly to level of testes. Forebody 58–224 (144), occupies 7.9–16.4% (12.9%) of body length. Oral sucker globular, 74–176 × 89–179 (132 × 151); thick post-oral collar present. Ventral sucker globular, slightly pedunculate (retracted in most specimens with peduncle visible as folds of tegument), with longitudinal aperture, 122–242 × 124–217 (181 × 176). Prepharynx observed occasionally (clearly visible in laterally mounted specimens), 10–32 (23) long. Pharynx rounded, similar size to oral sucker, 62–164 × 87–162 (107 × 134). Oesophagus short. Intestine bifurcates at level of ventral sucker. Caeca wide, extending posteriorly into hindbody, terminate blindly in posterior third of body. Testes sub-globular, separate, opposite, overlap caeca, occasionally overlap ovary and seminal receptacle, 67–196 × 70–176 (123 × 119). Cirrus-sac elongated oblong, thick-walled, overlaps ventral sucker sinistrally. Internal seminal vesicle bipartite, occupies three-quarters of cirrus-sac; anterior portion 57–97 × 29–47 (73 × 41); posterior portion 52–124 × 28–52 (85 × 44). Pars prostatica vesicular, 23–56 × 26–43 (36 × 35), occupies anterior quarter of cirrus-sac. Ejaculatory duct short. Genital pore sinistral, opens mid-way between oral and ventral suckers (at level of pharynx), 148–317 (207) from anterior end of body. Ovary sub-globular, median, at level of testes, overlaps seminal receptacle and occasionally ventral sucker dorso-anteriorly, 49–117 × 68–157 (82 × 114). Canalicular seminal receptacle reniform to oval, median, inter-testicular, 55–152 × 63–140 (115 × 117). Egg-forming complex not observed. Vitelline follicles irregularly globular, contiguous or slightly separated, predominantly extracaecal (some follicles overlap caeca), 29–72 × 28–65 (51 × 47), clustered in separate fields either side of ventral sucker, extend slightly into hindbody; vitelline fields with 8–11 follicles. Uterus occupies entire hindbody. Metraterm dorsal to ventral sucker and parallel to cirrus-sac. Eggs numerous, tanned, operculate, 34–44 × 16–23 (39 × 19). Excretory vesicle I-shaped, extends to level of caeca. Excretory pore terminal.

**Remarks**: *Innuptacola torquata* n. sp. differs from *I. gibsoni* by having vitelline follicles clustered laterally at the level of the ventral sucker, a slightly pedunculate ventral sucker in combination with caeca that extend beyond the level of the testes (*vs* in the anterior hindbody), and a proportionally larger seminal vesicle that occupies approximately three-quarters (*vs* two-thirds) of the cirrus-sac. In comparison with *L. pomacentri*, now the only pomacentrid-infecting species of *Lecithostaphylus, I. torquata* differs by having vitelline follicles that are clustered laterally at the level of the ventral sucker (*vs* in the hindbody surrounding the testes), and a bipartite, globular (*vs* unipartite and sinuous) seminal vesicle.

The post-oral collar in *I. torquata* is a distinct feature not reported for *I. gibsoni* (or species of *Lecithostaphylus*), but a comparable structure is seen in *Deretrema ludwicki*, which was described for specimens from *A. septemfasciatus* from the Seychelles. Comparisons between the present specimens of *I. torquata* and *D. ludwicki* are difficult given that the specimens of the latter were laterally flattened. However, based on the available description, *I. torquata* can be distinguished from *D. ludwicki* in the position of the vitelline follicles (clustered laterally at the level of the ventral sucker and extending slightly into the hindbody *vs* from the ventral sucker into the hindbody beyond the testes), and by the length of the oesophagus (short *vs* long). The illustration of *D. ludwicki* appears to show a pedunculate ventral sucker and bipartite seminal vesicle, which indicates that this species might be better placed in *Innuptacola*; examination of unflattened specimens of *D. ludwicki* from the type-host and locality are needed to confirm this.

Analyses of the novel molecular data for *Innuptacola torquata* demonstrate that it is widely distributed in the Central Indo-Pacific and has a broad, stenoxenous host-specificity. *Innuptacola torquata* has not yet been found in pomacentrids at Moreton Bay or New Caledonia, although sampling at these two localities is either relatively modest, with just 11 individuals from New Caledonia, and/or limited in diversity with collections comprising mostly species of *Abudefduf* at each locality.

Genus ***Deretrema*** Linton, 1910

#### *Deretrema stratiotes* n. sp. (Figure 6)

Type-host: *Abudefduf bengalensis* (Bloch), Bengal sergeant (Pomacentridae).

**Figure 6.**
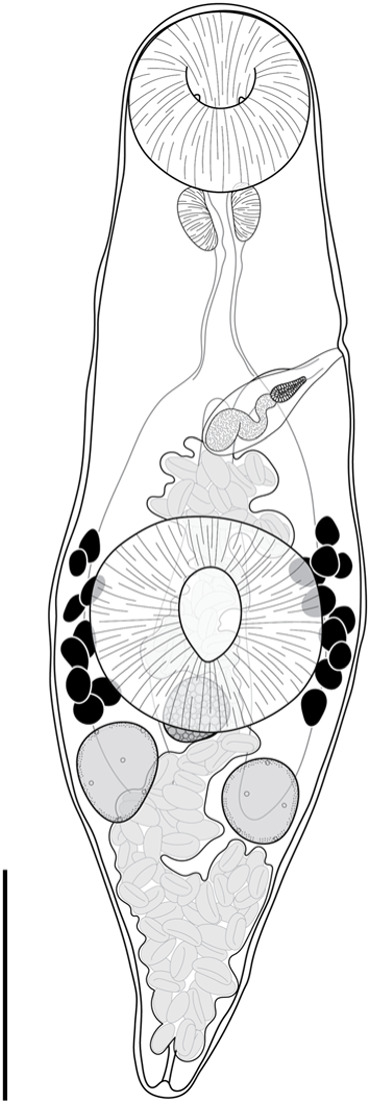
*Deretrema stratiotes* n. sp., holotype ex *Abudefduf bengalensis* from Ningaloo Reef. Scale bar = 200 *μ*m.

Type-locality: Norwegian Bay (22°36′S, 113°40′E), Ningaloo Reef, Western Australia, Australia.

Other hosts: *Abudefduf sexfasciatus* (Lacépède), Scissortail sergeant; *Abudefduf sordidus* (Forsskål), Blackspot sergeant; *Abudefduf vaigiensis* (Quoy & Gaimard), Indo-Pacific sergeant (Pomacentridae).

Other localities: Coral Bay (23°08′S, 113°46′E) and North West Cape (21°50′S, 114°01′E), Ningaloo Reef, Western Australia, Australia.

Abundance and prevalence: Norwegian Bay: two specimens from one of one (100%) *A. bengalensis*; three specimens from two of five (40%) *A. sexfasciatus*. Coral Bay: two specimens from two of two (100%) *A. bengalensis*; three specimens from three of seven (43%) *A. sexfasciatus*; two specimens from one of two (50%) *A. sordidus*; six specimens from four of four (100%) *A. vaigiensis*. North West Cape: one specimen from one of 11 (10%) *A. bengalensis*.

Site in host: Gall bladder.

Deposited material: Holotype (WAM V 12825) and 10 paratypes (WAM V 12826–12835).

Representative DNA sequences: ITS2 rDNA, five sequences (all submitted to GenBank, PQ571383–PQ571387); partial 28S rDNA, one sequence (submitted to GenBank, PQ571394); partial *cox*1 mtDNA, two sequences (both submitted to GenBank, PQ569605–PQ569606).

ZooBank Life Science Identifier: urn:lsid:zoobank.org:act:F4CCA921-6A0C-46C8-AE20-226509BDBE4F.

Etymology: The specific epithet ‘*stratiotes*’, based on the Greek noun στρατιώτης (*stratiṓtēs*) for soldier, refers to the collective common name of *Abudefduf* spp., the sergeant majors.

**Description**: [Based on ten whole mounted specimens and four hologenophores from all hosts.] Body narrow, lanceolate, widest at level of ventral sucker, 884–1469 × 262–600 (1164 × 378), tapered and rounded anteriorly, tapered and pointed posteriorly. Tegument covered with fine spines, most dense in pre-testicular region. Forebody 366–872 (545) long, occupies 41–46% (43%) of body length. Oral sucker globular, opening subterminally, with pair of papilla-like protrusions on internal surface, 103–223 × 115–232 (162 × 178). Ventral sucker rounded with longitudinal aperture, slightly wider than long, 169–302 × 172–360 (234 × 264). Prepharynx rarely discernible, dorsally overlaps posterior margin of oral sucker. Pharynx oval, 42–90 × 48–96 (68 × 71). Oesophagus 84–244 (141) long. Pharyngeal and oesophageal spines not detected. Intestine bifurcates in mid-forebody. Caeca blind, extend posteriorly to and dorsally overlap testes. Testes sub-globular, opposite, separated, in anterior third of hindbody, 63–154 × 59–135 (98 × 86). Cirrus-sac elongated oblong, thick-walled, in forebody, extends to level of intestinal bifurcation. Internal seminal vesicle unipartite, sinuous, 60–158 × 11–40 (115 × 32), occupies two thirds of cirrus-sac. Pars prostatica vesicular, occupies anterior third of cirrus-sac, 29–46 × 16–32 (37 × 23). Ejaculatory duct short. Genital pore sinistral, midway between oral and ventral suckers at level of intestinal bifurcation, 255–504 (324) from anterior end of body. Ovary subglobular, median, inter-caecal, overlaps posterior margin of ventral sucker dorsally, 54–159 × 62–169 (87 × 96). Canalicular seminal receptacle subglobular, immediately post-ovarian, 51–85 × 71–74 (68 × 71). Egg-forming complex not observed. Vitelline follicles irregularly globular, contiguous or slightly separated, 24–81 × 19–63 (44 × 37), clustered in separate fields either side of ventral sucker, span length of ventral sucker and extend into forebody, slightly overlap ventral sucker and caeca dorsally; vitelline fields with 9–12 follicles. Uterus occupies most of hindbody, extends into forebody and fills intercaecal space there. Metraterm parallel and dorsal to cirrus-sac. Eggs numerous, tanned, operculate, 24–42 × 15–19 (38 × 16). Excretory vesicle bulbous or tubular, extends anteriorly into mid-hindbody; lateral excretory arms extend into forebody, terminate midway between intestinal bifurcation and oral sucker, ventral to caeca, occasionally obscured by uterus. Excretory pore terminal.

**Remarks**: The specimens described here are consistent with the genus *Deretrema* as they possess a long oesophagus and globular vitelline follicles that are clustered laterally to the ventral sucker, and were found in the gall bladder of marine teleosts. *Deretrema stratiotes* n. sp. is the third species of the genus known from pomacentrid fishes. *Deretrema fusillus* was reported from *Abudefduf saxatilis* in the Dry Tortugas (Linton, 1910; Manter, 1934, 1947) and Puerto Rico (Dyer et al., 1985), and *D. ludwicki* was reported from *A. septemfasciatus* in the Seychelles (Toman, 1992). Toman and Kamegai (1974) reported an unidentified species of *Deretrema* from *A. sordidus* from the Mariana Islands but did not include a description. Although the site of infection for most species of *Deretrema* (including the new species described here) is the gall bladder, *D. fusillus, D. ludwicki* and the unidentified species of Toman and Kamegai (1974) were all reported to infect the intestine (Hanson, 1950; Toman and Kamegai, 1974; Toman, 1992); the original description of *D. fusillus* by Linton (1910) did not identify the site of infection. Additionally, four other species of *Deretrema*, from fishes other than pomacentrids, are also reported to infect the intestine: *D. acutum* Pritchard, 1963, *D. combesae* Bray & Justine, 2008, *D. combesorum* Bray & Justine, 2008, and *D. triodontis* Machida & Kuramochi, 1999.

*Deretrema stratiotes* does not closely resemble any previously described species of the genus. It differs from *D. acutum* by a longer oesophagus relative to body length (14% *vs* 7%), vitelline follicles that are typically distributed lateral to the ventral sucker (*vs* clustered in the posterior forebody), and caeca that terminate at the mid-level of the testes (*vs* posterior to the testes), and from *D. cholaeum* McFarlane, 1936 by having a unipartite (*vs* bipartite) seminal vesicle. *Deretrema stratiotes* differs from *D. combesae* by the extension of the cirrus-sac across the intestinal bifurcation (*vs* across the caeca posterior to the level of the bifurcation), and smaller suckers relative to testis size (oral sucker *vs* testis length × width, 1:0.69 × 0.55 *vs* 1:2.8 × 2.4; ventral sucker *vs* testis length × width, 1:0.48 × 0.36 *vs* 1:2.39 × 2.23), from *D. combesorum* by having smaller suckers relative to testis size (oral sucker *vs* testis length × width, 1:0.69 × 0.55 *vs* 1:1.96 × 1.34; ventral sucker *vs* testis length × width, 1:0.48 × 0.36 *vs* 1:1.8 × 1.1.1), and from *D. fellis* (Yamaguti, 1934) Yamaguti, 1940 by having a shorter hindbody relative to its body length (38% *vs* 56%). *Deretrema stratiotes* differs from *D. fusillus* by having more vitelline follicles (9–12 *vs* 6–8), from *D. ludwicki* by not having a post-oral collar and a narrower oesophagus, from *D. nahaense* and *D. pacificum* Yamaguti, 1942 by having a larger body to ventral sucker width ratio (1:0.62 *vs* 1:0.29 and 1:0.22, respectively), and from *D. ovale* Machida, 1984 by separate rather than confluent vitelline follicles. *Deretrema stratiotes* differs from *D. philippinense* Beverly-Burton & Early, 1982 by having testes in the hindbody (*vs* at the level of the ventral sucker), and a unipartite (*vs* bipartite) seminal vesicle, from *D. plotosi* Yamaguti, 1940 by a smaller sucker width ratio (1:1.53 *vs* 1:2.21), from *D. scorpaenicola* Bartoli & Bray, 1990 by a unipartite (*vs* bipartite) seminal vesicle, and from *D. sebastodis* (Yamaguti, 1934) Yamaguti, 1940 by having smaller sucker widths relative to testis widths (oral sucker *vs* testis width, 1:0.55 *vs* 1:1.3; ventral sucker *vs* testis width, 1:0.36 *vs* 1:0.81). *Deretrema stratiotes* differs from *D. triodontis* by having a unipartite (*vs* bipartite) seminal vesicle, a pars prostatica that occupies one quarter (*vs* half) of the cirrus sac, and vitelline follicles largely restricted to the ventral sucker zone (*vs* in the forebody to the level of the pharynx), and finally, from *D. woolcockae* Cribb, Wright & Bray, 1999 by having a shorter hindbody relative to body length (38% *vs* 50%) and a cirrus-sac that does not overlap the ventral sucker.

*Deretrema stratiotes* is so far known only from species of *Abudefduf* and only at Ningaloo Reef. This apparently strict stenoxenous host-specificity might be an artefact of sampling; only three other pomacentrid species have been examined at this locality: five *Dascyllus trimaculatus* (Rüppell), two *Neoglyphidodon melas* (Cuvier) and seven *Plectroglyphidodon obreptus* (Whitley). As species of *Abudefduf* have been sampled extensively at locations in the Pacific Ocean, especially in Queensland (over 500 individuals), it is likely that *D. stratiotes* is restricted to the Indian Ocean.

#### *Deretrema nahaense* (Yamaguti, 1942)

Type-host: *Thalassoma hardwicke* (Bennett), Sixbar wrasse (Labridae).

Type-locality: Naha, Japan.

Other records: Kamegai (1973); Cribb et al. (1999); Cribb et al. (2001); Olson et al. (2003); Bray et al. (2005); Muñoz et al. (2007).

*New material*

Known hosts: *T. hardwicke; Thalassoma lunare* (Linnaeus), Moon Wrasse (Labridae).

Known localities: Heron Island (23°26′S, 151°54′E), southern Great Barrier Reef, and Lizard Island (14°40′S, 145°26′E), northern Great Barrier Reef, Queensland, Australia.

Site in host: Gall bladder.

Deposited material: four vouchers (QM G241527–241530).

Representative DNA sequences: ITS2 rDNA, four sequences (three submitted to GenBank, PQ571388–PQ571390); partial 28S rDNA, one sequence (submitted to GenBank, PQ571395); partial *cox*1 mtDNA, four sequences (all submitted to GenBank, PQ569607–PQ569610).

**Description**: Yamaguti (1942); Cribb et al. (1999).

**Remarks**: The new material agrees with the descriptions by Yamaguti (1942) and Cribb et al. (1999). Although no sequence data are available for *D. nahaense* from the type-locality, there is no reason to suspect that the material from Australia does not represent this species.

## Discussion

### Recognition of Innuptacola

The new genus, *Innuptacola*, is recognized here on the basis of a combination of phylogenetic topology, host distribution and morphology. According to the key for the Zoogonidae of Bray (2008), both *I. gibsoni* and *I. torquata* are consistent with the genus *Lecithostaphylus*. The most common hosts for species of *Lecithostaphylus* are beloniform fishes (specifically from the families Belonidae, Exocoetidae, and Hemiramphidae), including the type-species, *L. retroflexus* (Molin, 1859) Odhner, 1911, proposed for specimens from *Belone belone* (Linnaeus) (as *B. acus* Risso). The analyses of the 28S rDNA dataset demonstrated that *L. brayi* and *L. halongi*, also from beloniform fishes, do not form a clade with *I. gibsoni* and *I. torquata* from pomacentrid fishes (Ovalentaria *incertae sedis*), with the two clades resolving as paraphyletic relative to *Deretrema* + *Proctophantastes*. Thus, host-specificity is seemingly a significant aspect of the distinction between *Lecithostaphylus* and *Innuptacola*. Morphologically, the new material differs from *Lecithostaphylus* on the basis of the seminal vesicle, which is distinctly bipartite in the new material and unipartite or sinuous in species of *Lecithostaphylus*. They also differ based on the vitelline follicle fields, which are typically lateral to the ventral sucker in the new material and post-ventral sucker in *Lecithostaphylus*. Although this distinction is not consistent for all individuals of *Innuptacola*, and can be dependent on the orientation of the specimens whereby the vitelline follicles may appear more or less clustered near the ventral sucker.

Based on the most recent key for the Lepidophyllinae *sensu* Blend et al. (2020) [which comprises all genera consistent with Lecithostaphylinae *sensu* Sokolov et al. (2021b)], *I. gibsoni* and *I. torquata* key to the genus *Whitegonimus* Jeżewski, Zdzitowiecki & Laskowski, 2009, which is recognized for a single species, *W. ozoufae* Jeżewski, Zdzitowiecki & Laskowski, 2009. Although there are no genetic data for *W. ozoufae*, it is clearly distinct from the new concept of *Innuptacola* in that it has an I-shaped or saccular *vs* Y-shaped excretory vesicle, oblique *vs* tandem testes, short *vs* long genital atrium/ejaculatory duct, uneven *vs* equal number of vitelline follicles in each field, and caeca reaching beyond *vs* to the level of the testes. The two concepts are also clearly distinct in terms of host range and geographic distribution; with *W. ozoufae* described from a nototheniid off the coast of Argentina and Chile in the eastern Pacific Ocean (Jeżewski et al., 2009, 2014; Muñoz, 2020).

### Status of the genus Lecithostaphylus Odhner, 1911

With the recombination of *I. gibsoni*, there are now 12 species that are putatively referable to the genus *Lecithostaphylus: L. brayi; L. buckleyi* (Ramadan, Morsy & Lashein, 2003); *L. depauperati* Yamaguti, 1970; *L. fugus* Zhang, Qiu & Li, 1986; *L. halongi; L. hemiramphi* (Manter, 1947) Yamaguti, 1971; *L. ismailensis* Ramadan, Morsy & Lashein, 2003; *L. nitens* (Linton, 1898) Linton, 1940; *L. parexocoeti* (Manter, 1947) Yamaguti, 1971; *L. pomacentri; L. retroflexus*; and *L. tylosuri* Châari, Derbel & Neifar, 2013. On the basis of morphological and host distinctions, three of these are considered inconsistent with *Lecithostaphylus* or any other presently recognized zoogonid genus: *L. buckleyi, L. fugus*, and *L. pomacentri*.

*Lecithostaphylus buckleyi*, from the intestine of a dorosomatid fish, *Sardinella gibbosa* (Bleeker), was described as having testes in the posterior hindbody and the vitelline follicles as numerous and extending from the intestinal bifurcation in the mid-forebody to the posterior hindbody (Ramadan et al., 2003). These features are distinct from other *Lecithostaphylus* species in which both the testes and vitelline follicles are predominantly in the anterior hindbody to mid-hindbody. Based on these morphological distinctions and the host identity (Clupeiformes *vs* Beloniformes), *L. buckleyi* is not a convincing member of this genus, and is considered here a *species inquirendum*.

*Lecithostaphylus fugus* appears to be consistent with the fellodistomid genus *Lintonium* Stunkard & Nigrelli, 1930. *Lecithostaphylus fugus* shares with *Lintonium* spp. the two lateral columns of post-ovarian vitelline follicles, the median genital pore, the lobed ovary and the uterus being entirely intercaecal (Cribb et al., 2021). In addition, *Lec. fugus* was described based on specimens from the intestine of a tetraodontiform fish, *Takifugu niphobles* (Jordan & Snyder) [as *Fugus niphobles* (Jordan & Snyder)], which is consistent with the tetraodontiform hosts of *Lintonium* spp. (see Bray, 2002; Cribb et al., 2021). Comparison of this species with existing species of *Lintonium* is inconclusive, as the taxonomy of *Lintonium* itself requires further work (see Cribb et al., 2021). To facilitate future studies on the genus, *Lec. fugus* is here recombined as *Lintonium fugus* (Zhang, Qiu & Li, 1986) n. comb. However, it is plausible that this species will prove to be a junior synonym of an existing species of the genus.

The description of *Lecithostaphylus pomacentri* was based on a seemingly distorted specimen from the intestine of a pomacentrid fish, *Neopomacentrus taeniurus* (Bleeker) [as *Pomacentrus taeniurus* Bleeker (see Toman, 1992)]. As described, it differs dramatically from other species of *Lecithostaphylus* and *Innuptacola*, in that it possesses just a single field of six large vitelline follicles, and appears to have a median genital pore. On the basis of this morphological distinction, *Lec. pomacentri* is here considered a *species inquirendum*.

On the basis of these interpretations, the genus *Lecithostaphylus* now comprises nine species and the generic diagnosis of Bray (2008) can be refined: ‘seminal vesicle elongate or winding’ and ‘parasites principally of beloniform fishes’. Undoubtedly, these suggestions/hypotheses will require testing using molecular data generated from samples collected from the type-host and/or -locality.

### The Zoogonidae

Until recently, the Zoogonidae comprised three subfamilies: the Cephaloporinae, the Lepidophyllinae and the Zoogoninae (Cutmore et al., 2014; Blend et al., 2020). Recent molecular work showed that species from three lepidophylline genera are relatively distantly related, resulting in the resurrection of the Lecithostaphylinae, into which all but two lepidophylline genera were transferred (Cabañas-Granillo et al., 2020; Sokolov et al., 2021b). The analyses in the present study included representatives from all four subfamilies: one cephaloporine genus, five lecithostaphyline genera (including *Innuptacola*), one lepidophylline genus and three zoogonine genera, and generally support the subfamilial divisions, with the exception of the position of a lecithostaphyline species, *Steganoderma* cf. *eamiqtrema*, which resolved relatively distant to other lecithostaphyline taxa, consistent with the findings of Sokolov et al. (2021b). However, it should be noted that the sequence of *Steganoderma* cf. *eamiqtrema* was generated from metacercariae which were identified on the basis of morphological similarities to the adult specimens described by Blend and Rácz (2020), and the identity of the intermediate host [which for this genus are known to be species of *Chionoecetes* Krøyer (Oregoniidae) based on previous studies by Kagei and Kon (1978) and Ryazanova (2018)]. Further work is certainly needed to justify division of the Lecithostaphylinae. The phylograms produced by the analyses in the present study suggest that the Zoogonidae is paraphyletic relative to the Faustulidae *sensu lato*, consistent with the findings of Hall et al. (1999), Olson et al. (2003), Cutmore et al. (2014), Sokolov et al. (2016), Pérez-ponce de León and Hernández-Mena (2019), Cabañas-Granillo et al. (2020), Sokolov et al. (2021b) and Atopkin et al. (2022).

### Biogeography and interpretations of molecular barcode data over range

*Deretrema stratiotes*, found here only at Ningaloo Reef, likely does not occur in eastern Australian waters, even though all four known host species (all *Abudefduf* spp.) have broad distributions in the Indo-West Pacific, spanning at least the entire Central Indo-Pacific region. This interpretation is based on substantial sampling of *Abudefduf* spp. on the Great Barrier Reef and in Moreton Bay (>500 individuals). In contrast, both species of *Innuptacola* were detected from both eastern and western Australian waters, with *I. gibsoni* detected as far east as New Caledonia and *I. torquata* as far as French Polynesia.

Indeed, the distribution of *I. torquata* is among the most widespread for digeneans of Indo-West Pacific coral reef fishes which have been corroborated by genetic data. Another example of a fish-trematode distribution from Ningaloo Reef to French Polynesia, that is corroborated by genetic data, is that of *Preptetos laguncula* Bray & Cribb, 1996 (Lepocreadiidae) in acanthurids (Bray et al., 2022). The distribution of *Elaphrobates chaetodontis* (Yamaguti, 1970) Yong, Cribb & Cutmore, 2021 (Aporocotylidae) is comparable, in chaetodontids, and is supported by molecular data from the Great Barrier Reef to French Polynesia and north to Okinawa (Cutmore and Cribb, 2022). Two distributions supported by molecular data exceed that of *I. torquata*: that of *Gorgocephalus yaaji* Bray & Cribb, 2005 (Gorgocephalidae) in kyphosids from the Great Barrier Reef east to French Polynesia but also south to off Kioloa, New South Wales and west to Sodwana Bay, South Africa (Huston et al., 2016, 2021), and *Schikhobalotrema acutum* (Linton, 1910) Skrjabin & Guschanskaja, 1955 (Haplosplanchnidae) in belonids from Queensland waters and the Gulf of Mexico (Pérez-ponce de León et al., 2024). Critically, the scarcity of such examples does not necessarily imply that most trematodes of Indo-West Pacific coral reef fishes have more restricted distributions, as the overwhelming majority of sequence data have been generated from taxa collected in Queensland waters (Cribb et al., 2016).

Although we consider inclusion of molecular analyses essential to demonstrate broad distributions with confidence, interpretation of small genetic differences over range is difficult. Here, for *I. torquata*, the ITS2 rDNA sequences are identical across its distribution, whereas the *cox*1 mtDNA sequences formed three clades differing at 10–35 bp, one each for Ningaloo Reef, the Great Barrier Reef and French Polynesia. Relative to the above comparable examples, this level of intraspecific variation is low: for *Preptetos laguncula* and *Gorgocephalus yaaji*, reported intraspecific variation between biogeographically distinct Indo-West Pacific localities in ITS2 is 1–3 and 0–5 bp, and in *cox*1 6–54 and 12–62 bp, respectively (Huston et al., 2021; Bray et al., 2022). It is important to emphasise that comparison against these values is not sufficient to determine whether variation over range should be interpreted as interspecific or intraspecific; in each example above, as here, conclusions of conspecificity over range have been determined based on an integrated consideration of evidence.
